# A Preliminary Exploratory Study Based on LC–MS/MS and LFQ for Candidate Protein Biomarkers in Peripheral Blood from Rheumatoid Arthritis

**DOI:** 10.3390/biomedicines14102182

**Published:** 2026-09-26

**Authors:** Maria P. Toussaint-Padilla, Sherine Gabriel, Timothy L. Karr, Efraín Flores-Hernández, Arnulfo Hernán Nava-Zavala, Erika A. Martínez García, Maria G. Zavala-Cerna

**Affiliations:** 1 Immunology Research Laboratory, Decanato Medicina, Universidad Autónoma de Guadalajara, Av. Patria 1201, Lomas del Valle, Zapopan 45129, Jalisco, Mexico; maria.toussaint@edu.uag.mx; 2 Fulton Center, ASU Health, 300 East University Drive, Tempe, AZ 85287, USA; sherine.gabriel@asu.edu; 3 Biosciences Mass Spec/Proteomics Core, Arizona State University, 1001 S. McAllister Ave, Tempe, AZ 85281, USA; tkarr@asu.edu; 4 Unidad Académica Ciencia y Tecnología, Universidad Autónoma de Guadalajara, Av. Patria 1201, Lomas del Valle, Zapopan 45129, Jalisco, Mexico; efrain.flores@edu.uag.mx; 5 International Program of Medicine, Universidad Autonoma de Guadalajara, Av. Patria 1201, Lomas del Valle, Zapopan 45129, Jalisco, Mexico; arnulfo.nava@edu.uag.mx; 6 Departamento de Inmunología y Reumatología del Hospital General de Occidente, Secretaría de Salud Jalisco, Guadalajara 45170, Jalisco, Mexico; 7 Departamento de Biología Molecular y Genómica, Instituto de Investigación en Reumatología y del Sistema Músculo Esquelético, Centro Universitario de Ciencias de la Salud, Universidad de Guadalajara, Guadalajara 44340, Jalisco, Mexico; eaurora.martinez@academicos.udg.mx

**Keywords:** k exploratory study, rheumatoid arthritis, proteomics, biomarkers, mass spectrometry, autoimmunity

## Abstract

**Objective:** Detangling the rheumatoid arthritis (RA) proteomic profile is of interest to improve diagnosis, RA subclassifications, and targeted treatment. We aimed at identifying serum proteins as candidate biomarkers for RA. **Methods:** This case-control study included participants with RA and healthy controls (HCs) paired by gender; clinical information and proteomic profiles were analyzed for the identification of candidate protein biomarkers. A statistical analysis included logistic regression and ROC curves for AUC measurement. **Results:** Following the screening of 176 patients, 25 RA and 25 HC patients were included in this study. The average age was 51 ± 12 years, and 90% were females. Notably, we identified five serum protein candidate biomarkers with differential expression when comparing RA to HC, consisting of testis-expressed protein 13B (*p* = 6.31 × 10^−4^), keratin type II cytoskeletal 79 (*p* = 1.317 × 10^−4^), 60 kDa heat shock protein (*p* = 7.594 × 10^−4^), pseudo uridylate synthase 7 homolog (*p* = 8.332 × 10^−3^), and SUN domain-containing protein 3 (*p* = 1.883 × 10^−7^). The first four proteins were predominantly observed in patients with RA, while the last was predominantly identified in the HCs. **Conclusions:** A distinctive serum candidate biomarker profile consistent in five proteins was identified for RA, suggesting a unique stress-related, inflammatory signature. Collectively, the profile exhibits potential, but our findings must be validated in larger cohort studies, preferably in the context of undifferentiated arthritis before a definitive model for diagnosis is established.

## 1. Introduction

Rheumatoid arthritis (RA) is a chronic and progressive systemic autoimmune disease characterized by erosive arthritis of small joints predominantly, systemic inflammation, and decreased life expectancy [1]. The RA prevalence range is estimated to be from 0.5 to 1% worldwide. However, it might be increasing according to previous information, from a trend of 0.62% in 1995 to 0.72% in 2007 in some regions [2]. Due to the undulant course of the disease, diagnosis can be challenging. Traditionally, the classification criteria, which were updated in 2010 by the American College of Rheumatology (ACR)/European League Against Rheumatism (EULAR), are used for research purposes, although routine use is frequent in rheumatology clinics. The primary update for this classification criteria focuses on features that appear during early stages of the disease, such as a positive result for anti-citrullinated peptide antibodies (anti-CCP) [3]. Included in the classification criteria is also the rheumatoid factor (RF), with even more limited specificity (70–85%) than anti-CCP (90–99%) for RA; however, the positive rate of RF in RA patients is reported to be similar for both tests and ranges from 60 to 75% [4]. This suggests that there is room to improve the diagnostic efficacy, probably pointing towards the reason why they are not considered as standalone tests for the diagnosis of RA. Additionally, these tests cannot distinguish patients with high or low disease activity [5]. Early diagnosis followed by prompt treatment has been shown to substantially diminish the risk of joint destruction and patients’ disability [6].

However, up to one third of patients will not be classified as having RA, especially during early stages and in periods of low disease activity. These unclassified subgroups or seronegative patients are vulnerable to continuing with the natural course of the disease and subject to increased activity and later disability, which underlines the need for additional biomarkers. Furthermore, it is of utmost importance that the diagnosis can direct the treatment in a personalized form based on subject vulnerabilities and in a timely manner [7]. Compared to other techniques that address transcription, proteomic analysis can include post-transcriptional modifications of proteins, as well as their interactions with tissues [8], which is an added value for novel biomarkers. Previous proteomic studies with high-resolution mass spectrometry (MS) in RA patients have identified peripheral proteins associated with the disease, identifying Ficolin 2 (FCN-2) correlated to the number of swollen joints and disease activity score [9]. A second study, based on MS proteomics and a random forest machine learning model to predict protein–protein interactions, found 24 protein biomarkers; out of those, both orosomucoid 1 (ORM1) and haptoglobin (HP) exhibited a positive correlation with disease activity, and their presence yielded a diagnostic value of >0.75 (AUC) [10].

Although the identification of new biomarker candidates for RA seems promising, these findings are less informative, as they are missing relevant clinical context such as disease duration, disease activity, and other key parameters related to the natural history of the disease. Most studies that try to address the identification of biomarkers fail to classify patients clinically, making it difficult to draw inferences for patient care.

To address this, we ought to search for candidate biomarkers using MS-based proteomics in RA patients and healthy controls (HCs). As secondary objectives, we searched for associations between candidate protein biomarkers and anti-CCP status, disease activity, acute phase reactants, and demographic confounders.

## 2. Materials and Methods

### 2.1. Study Population

This was a case-control study, conducted between January and June 2024. The study was written in accordance with STARD guidelines for reporting diagnostic accuracy studies [11].

Participants were invited and informed consent was signed prior to enrollment in our study. Cases were defined as consecutive subjects who fulfilled classification criteria for RA according to the 2010 American College of Rheumatology/European League Against Rheumatism (ACR/EULAR) criteria [3] and who attended a rheumatology clinic in a third-level referral hospital. Participants were invited if they met other inclusion criteria such as being 18 years or older and having a disease duration of at least 6 months with no change in medications in the previous 3 months to the collection of the sample.

Controls were selected from consecutive blood donors who voluntarily attended the blood bank of that same institution and denied having any chronic transmissible and non-transmissible diseases; the latter was corroborated with routine exam laboratories performed at the blood bank.

Controls were paired to RA participants by gender; their clinical information was obtained through clinical interview and chart review. Physical examination and specific measurements about disease activity and disability were obtained by a rheumatologist with DAS-28 [12] and HAQ–DI indexes [13], respectively.

Exclusion criteria were patients with other rheumatological conditions or mixed rheumatic diseases, and patients or controls with loss of serum integrity, which was estimated by total protein count, before the proteomic analyses. HCs were also excluded if they had a positive HB antigen, DNA-HBV, anti-HCV, anti-HIV, or antigen HIVRNA-HIV.

### 2.2. Sample Collection and Laboratory Measurements

Peripheral blood samples were obtained by venipuncture, allowed to clot for 30 min at room temperature, and centrifuged at 3000 rpm for 10 min to separate serum. Aliquots were stored at −80 °C until proteomic processing. Laboratory measurements were performed by personnel who were blinded to disease status.

### 2.3. ELISA

The measurement of anti-CCP antibodies for both cases and controls was made using a commercially available second-generation enzyme-linked immunosorbent assay, according to the manufacturer’s instructions (Axis-Shield Dundee, Scotland, UK). Briefly, 50 μL of calibrators, control samples, and patient samples were incubated into appropriate wells with the addition of a buffer. Then, microtiter plates were coated with 100 μL of enzyme conjugate (peroxidase anti-human-IgG) for 1 h at 37 °C and washed three times. A chromogenic substrate (TMB) was added to each well and left for 30 min. The reaction was stopped (sulfuric acid 0.25 mmol/L), and the monochromatic measurement was performed at 405/620 nm in a spectrometer (Thermo Scientific^®^ Waltham, MA, USA). Results were reported in OD and then converted to units per milliliter (U/mL); a result > 5 U/mL was considered positive.

### 2.4. Sample Preparation for Proteomics

Serum samples were thawed on ice and centrifuged at 12,000× *g* for 10 min at 4 °C to clear insoluble lipids and particulate matter. Total protein concentration was determined using the Bradford assay according to the manufacturer’s protocol. To enrich for lower-abundance candidate biomarkers, high-abundance serum proteins were removed using top-14 immunodepleting spin columns (Agilent Technologies, Santa Clara, CA, USA) following the manufacturer’s instructions, followed by buffer exchange into 50 mM ammonium bicarbonate using 3 kDa molecular weight cut-off centrifugal filters (Amicon Ultra, Millipore, Darmstadt, Germany). Depleted serum samples were denatured in 6 M urea, reduced with 10 mM dithiothreitol (DTT) for 45 min at 56 °C, and alkylated with 20 mM iodoacetamide (IAA) in the dark for 30 min at room temperature. Proteins were digested with sequencing-grade modified trypsin (Promega, Wisconsin, USA) at a 1:50 (*w*/*w*) enzyme-to-protein ratio overnight at 37 °C. Digestion was quenched by acidifying the reaction with 0.1% trifluoroacetic acid (TFA, *v*/*v*). The resulting peptide mixture was desalted using C18 solid-phase extraction cartridges, dried under vacuum in a SpeedVac, and reconstituted in 0.1% formic acid in LC–MS-grade water prior to liquid chromatography–tandem mass spectrometry (LC–MS/MS) analysis.

### 2.5. Liquid Chromatography–Tandem Mass Spectrometry

All LC–MS analyses were performed at the Biosciences Mass Spectrometry Core Facility (https://cores.research.asu.edu/mass-spec/) at Arizona State University (Date: 20 March 2025). All data-dependent mass spectra were collected in positive mode using an Orbitrap Fusion Lumos mass spectrometer (Thermo Scientific) coupled with an UltiMate 3000 UHPLC (Thermo Scientific). One µL of peptides was fractionated using an Easy-Spray LC column (50 cm × 75 µm ID, PepMap C18, 2 µm particles, 100 Å pore size, Thermo Scientific) equipped with an upstream 300 μm × 5 mm trap column. Electrospray potential was set to 1.6 kV and the ion transfer tube temperature was set to 300 °C. The mass spectra were collected using the “Universal” method optimized for peptide analysis provided by Thermo Scientific. Full MS scans (375–1500 *m*/*z* range) were acquired in profile mode with the Orbitrap set to a resolution of 120,000 (at 200 *m*/*z*), cycle time set to 3 s, and mass range set to “Normal”. The RF lens was set to 30% and the AGC set to “Standard”. Maximum ion accumulation time was set to “Auto”. Monoisotopic peak determination (MIPS) was set to “peptide” and included charge states 2–7. Dynamic exclusion was set to 60 s with a mass tolerance of 10 ppm and the intensity threshold set to 5.0 × 10^3^. MS–MS spectra were acquired in a centroid mode using quadrupole isolation window set to 1.6 (*m*/*z*). Collision-induced fragmentation (CID) energy was set to 35% with an activation time of 10 milliseconds. Spectra were acquired over a 180 min gradient and a flow rate of 0.250 μL/min, as follows: 0–10 min at 0–2%, 10–100 min at 2–8%, 100–110 min at 8–32%, 110–120 min at 32–35%, 120–125 min at 35–45%, 125–155 min at 45–98%,155–165 min at 98%, and 165–180 min at 2%, followed by a post-run wash step.

### 2.6. Label-Free Quantification (LFQ)

We analyzed raw files searched against the Uniprot (www.uniprot.org) Homo sapiens (sp_canonical TaxID = 9606_and_subtaxonomies) (v2023-09-13) using Proteome Discover 3.2 (Thermo Scientific). Raw files were searched using SequestHT that included trypsin as the enzyme, three maximum missed cleavage sites, min–max peptide length of 6/144, precursor ion (MS1) mass tolerance set to 20 ppm, fragment mass tolerance set to 0.5 Da, and a minimum of one peptide identified. Carbamidomethyl (C) was specified as fixed modification, and dynamic modifications were set to Acetyl and Met-loss at the N-terminus and oxidation of Met. A concatenated target–decoy strategy and a false-discovery rate (FDR) set to 1.0% were calculated using Percolator [14]. The data were imported into Proteome Discoverer 3.2 (Thermo Scientific Inc. Waltham, MA, USA), and accurate mass and retention time of detected ions (features) were taken using Minora Feature Detector algorithm in Proteome Discoverer. The identified Minora features were then used to determine area-under-the-curve (AUC) of the selected ion chromatograms of the aligned features across all runs and relative abundances calculated.

### 2.7. Data Analysis

For descriptive statistics, we used mean and standard deviation for variables with normal distribution. Abnormally distributed variables were presented with median and interquartile range (IQR). Categorical variables were reported as frequencies and percentages. For inferences in antibody titers and other characteristics of patients, we used the independent t-test to compare quantitative variables and Chi-square test to compare qualitative variables.

Biomarker discovery and feature prioritization were primarily conducted using a regularized multivariate machine learning architecture (L2-penalized logistic regression within a nested cross-validation scheme) to assess joint feature contributions while mitigating overfitting.

To provide descriptive univariate characterization between rheumatoid arthritis (RA) and healthy control (HC) cohorts, two-sided Mann–Whitney U tests were performed. To account for multiplicity across the 735 simultaneously quantified proteins, raw *p*-values were adjusted using the Benjamini–Hochberg False Discovery Rate (FDR) method. Both nominal *p*-values and FDR-adjusted q-values are reported. Fold-change values were computed as the ratio of mean abundances (RA:HC). Statistical analyses and multiple-testing corrections were executed in Python v3.10.6 using SciPy v1.15.3, and Statsmodels v0.14.6. Feature transformation, multivariable regularized logistic regression, and cross-validation were implemented via scikit-learn v1.7.2.

## 3. Results

**Figure 1:**
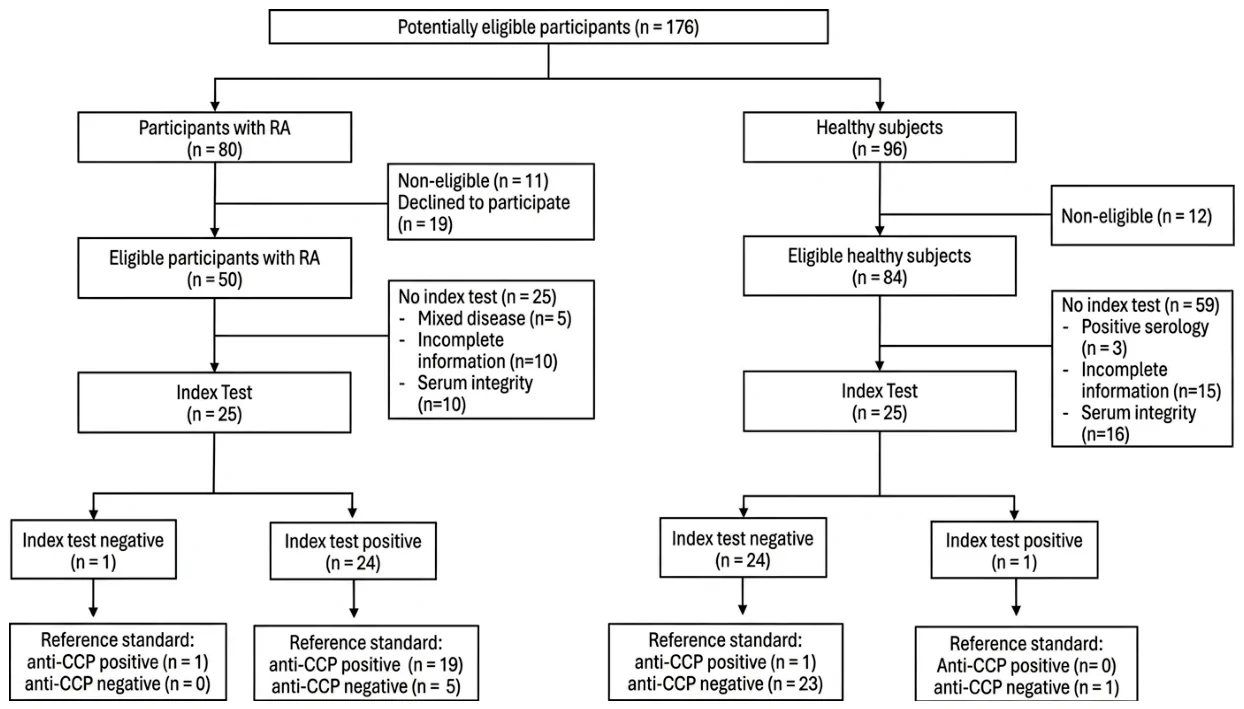
STARD diagram to report flow of participants through the study. The index test refers to the proteomic profile.

A total of 176 subjects for participation were screened; eligible cases were 50 for RA and 84 for HC (Figure 1). The index test (identification of a panel of serum biomarkers) was performed in 25 HC and 25 RA patients with a mean ± SD age of 50.8 ± 11.7, and 90% were women. The characteristics for each group are represented in Table 1.

As a reference test for RA, we performed a measurement of anti-CCP antibodies, and as expected, titers were significantly elevated in RA compared to HC, with a median (ICR) of 23.5 (5.6–106.3) and 4.4 (4.4–4.6), respectively (*p* < 0.0001).

To investigate the discriminatory capacity of circulating serum proteins between patients with rheumatoid arthritis (RA) and healthy controls (HCs), we profiled the serum proteome across all quantified proteins (*n* = 735). Feature prioritization was performed by training a regularized multivariable logistic regression model on standardized abundance profiles. Candidate biomarkers were ranked by the absolute magnitude of their regression coefficients (∣β∣), capturing their conditional contribution to class separation while accounting for inter-feature covariance.

Table 2 summarizes the top 10 discriminative proteins identified by the model, alongside univariate non-parametric statistics (two-sided Mann–Whitney U test) and Benjamini–Hochberg false discovery rate (FDR) adjustments across all 735 proteins. Among these 10 candidates, six exhibited statistically significant differential abundance at FDR < 0.05, two demonstrated marginal significance (FDR < 0.10), and two (PUS7 and LOR) exhibited FDR > 0.10. Despite its moderate univariate significance (raw *p* = 0.0083, FDR = 0.1094), PUS7 ranked third in multivariable importance (∣β∣ = 0.0182), reflecting its complementary discriminatory value in resolving sample heterogeneity when modeled jointly with high-confidence markers such as SUN3 and HSP60. Consequently, the top five features were prioritized for downstream low-dimensional classification modeling.

**Figure 2:**
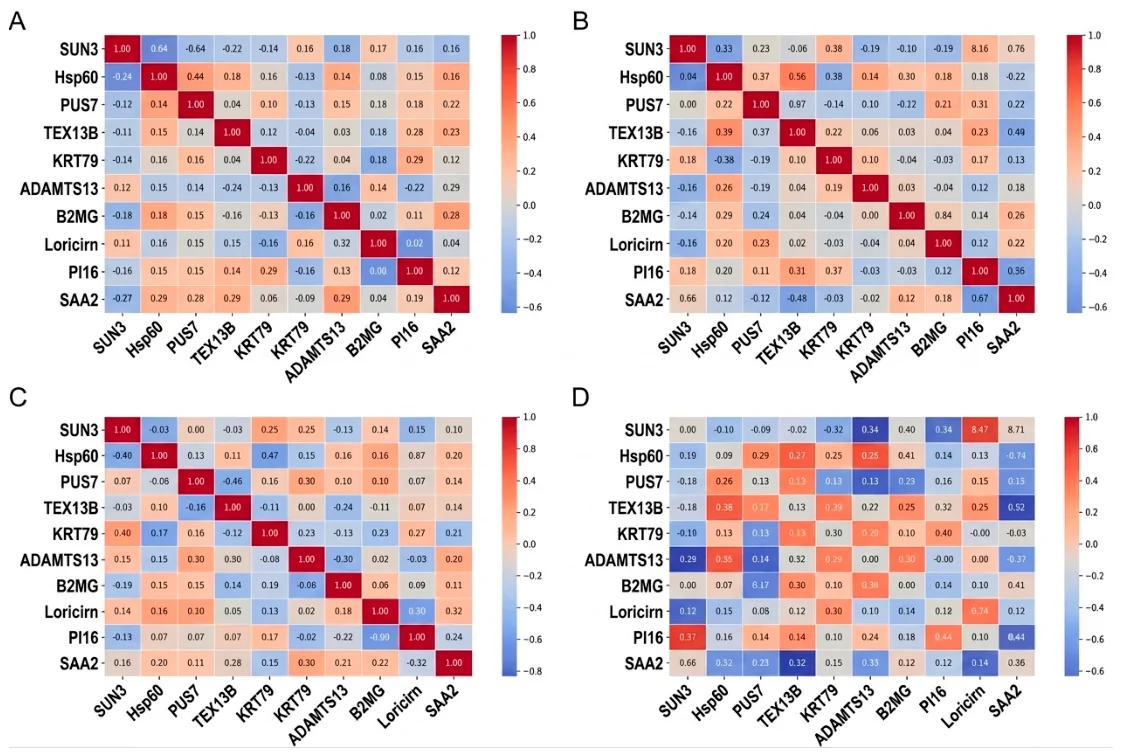
Heatmaps showing Pearson correlation coefficients among the top 10 proteins identified in the study across four different subsets: (**A**) All participants, (**B**) Rheumatoid Arthritis (RA) patients, (**C**) Healthy controls (HC), and (**D**) RA vs. Control comparison. Each cell represents the degree of correlation (r) between expression levels of two proteins, with red indicating positive correlation and blue indicating negative correlation. Distinct patterns emerge between subgroups, highlighting potential co-regulation or inverse expression relationships in RA.

We then analyzed by the creation of heatmaps the correlation of these ten proteins in four different scenarios: a correlation heat map for all study participants, correlation heat maps for RA patients, a correlation heat map for HC, and a final correlation heat map for differences between RA and HC (Figure 2A–D, respectively). 

**Figure 3:**
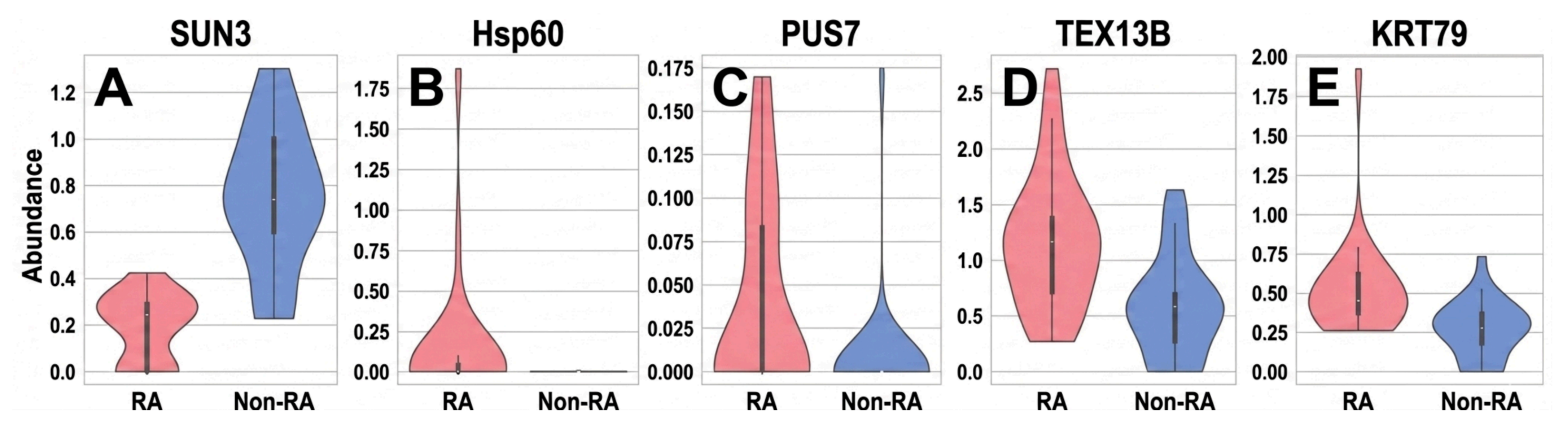
Violin plots showing the expression distribution of the five most significantly differentially expressed proteins between rheumatoid arthritis (RA, *n* = 25) and healthy control (HC, *n* = 25) groups. Each panel (**A**–**E**) represents the differential expression of the five more distinctive variations in protein expression: (**A**) SUN domain-containing protein 3 (SUN3) (*p* = 0.0000), (**B**) 60 kDa heat shock protein (Hsp60) (*p* = 0.0008), (**C**) Pseudo uridylate synthase 7 homolog (*p* = 0.0083), (**D**) Testis-expressed protein 13B (TEX13B) (*p* = 0.0006), and (**E**) Keratin type II cytoskeletal 79 (KRT79) (*p* = 0.0001). Expression values were log-transformed and normalized using the interquartile range. Red violins correspond to RA patients and blue violins to HC subjects. Each dot represents an individual sample.

Five prioritized candidate biomarkers, namely SUN domain-containing protein 3 (SUN3) (Q8TAQ9), were found to be positive in 17 subjects with RA and in all controls, suggesting a possible suppressive or regulatory role in the disease state. The 60 kDa heat shock protein (Hsp60) (P10809) was found in 11 RA samples and only one HC sample, tRNA pseudo uridylate synthase 7 (PUS7) (Q96PZ0) was found in nine RA samples and one HC sample, testis-expressed protein 13B (TEX13B) (Q9BXU2) was found in all 25 RA samples and 19 HC samples, and keratin type II cytoskeletal 79 (KRT79) (Q5XKE5) was found in 25 RA samples and 21 HC samples. These five proteins were successfully integrated into a robust predictive model capable of effectively detecting the presence of RA. A comparative analysis of the expression levels of these five proteins is shown in Figure 3.

Importantly, the five proteins carry largely independent information since their pairwise correlations were all weak (strongest around 0.31), and collinearity was below 1.3, suggesting that they can be placed in the same model.

Logistic regression was performed using protein expression values as features to further quantify each protein’s predictive contribution to RA classification. As shown in Table 3, HSP60, PUS7, TEX13B, and KRT79 had positive regression coefficients, suggesting that their increased expression was positively associated with RA status. In contrast, SUN3 exhibited a negative coefficient, where a one-standard-deviation rise in SUN3 cuts the odds of belonging to the RA group by about 99%, while a comparable rise in KRT79 multiplies those odds by roughly nine-fold. In our study, the included healthy controls were significantly younger than the other cases, which was expected given the fact that blood donors tend to be younger compared to the age of diagnosis for RA patients. A regression analysis for age in our study suggested that every 12 years of age raises the odds by about four-fold. The HSP60-related estimates and the anti-CCP levels are so extreme because those variables almost perfectly split the two groups.

To combine protein analysis with additional variables, three multi-variable models were built: one using only the five candidate protein biomarkers (A1 and A2), a second one adding sex and age (B1) (given the differences in age on both groups), and a third model adding anti-CCP status (C1) (Table 4). Because the healthy group was not given disease-activity or routine laboratory workups, only age, sex, anti-CCP status, and the abundance of proteins were included. Cross-validated AUC (repeatedly holding data out) is the fairer performance measure than the in-sample apparent AUC.

**Figure 4:**
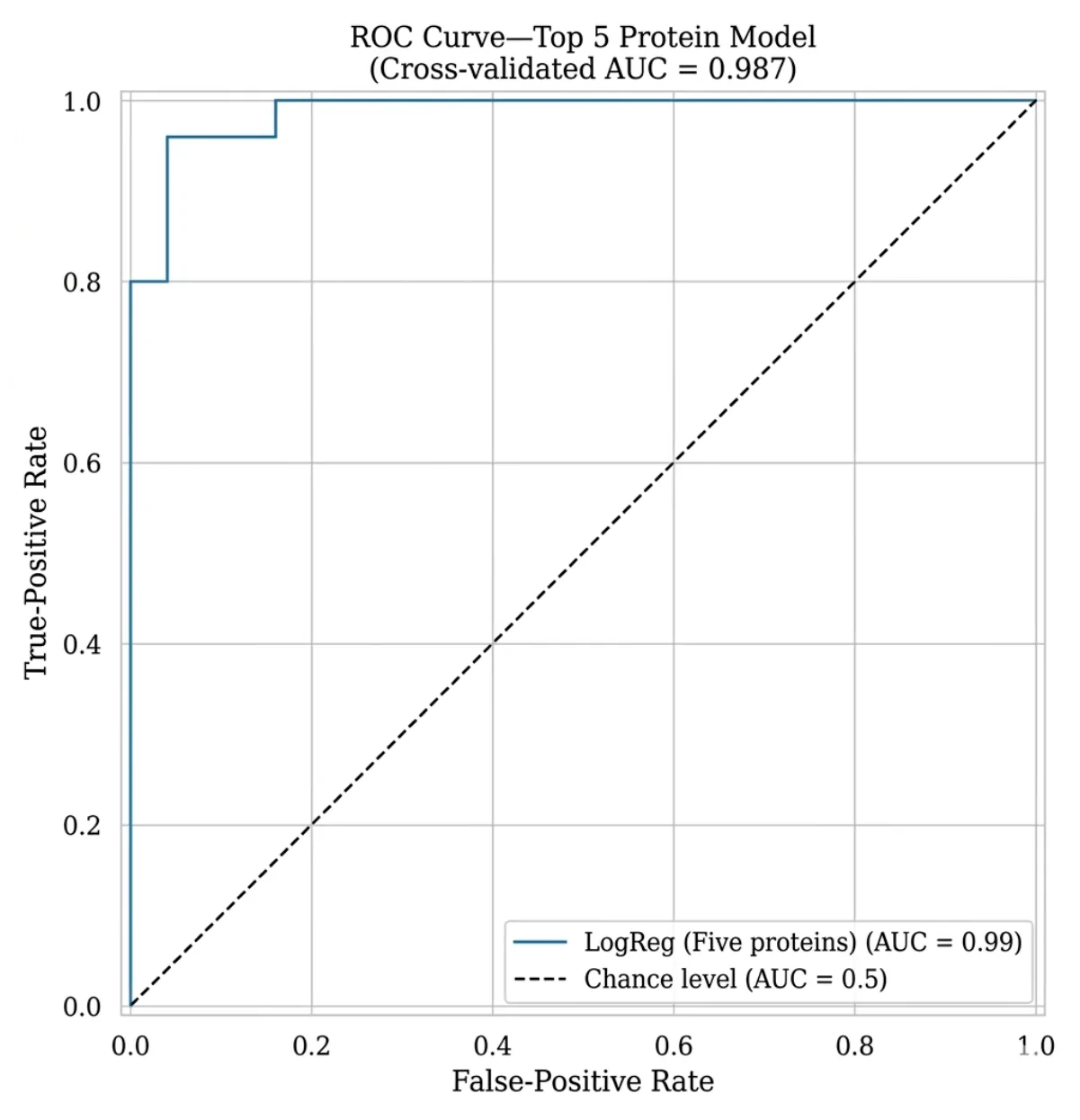
ROC curve of top five differentially expressed proteins: HSP60 (P10809), PUS7 (Q96PZ0), TEX13B (Q9BXU2), KRT79 (Q5XKE5), and SUN3 (Q8TAQ9).

After multivariable logistic regression models, the five-protein panel (TEX13B, SUN3, KRT79, HSP60, and PUS7) showed strong discriminatory capacity for RA versus HC, with cross-validated AUC values close to 0.98 (Figure 4). This indicates that the combined candidate biomarker signature retained high predictive performance even when the contribution of the individual proteins was considered simultaneously. Higher TEX13B, KRT79, HSP60, and PUS7 abundance were associated with an increased probability of RA, whereas higher SUN3 abundance was associated with a lower probability of RA. When age was incorporated into the model, overall predictive performance remained essentially unchanged, suggesting that the protein signature captures substantial information beyond the age difference observed between RA patients and HC. Although age was strongly associated with RA in the univariable analysis, its adjusted effect was attenuated after inclusion of the candidate protein biomarkers, with wide confidence intervals and lack of statistical significance. This attenuation suggests that part of the apparent age-related risk may overlap with, or be reflected by, the molecular profile represented by the protein panel. Importantly, this does not imply that age is clinically irrelevant; rather, within this small dataset, the proteins appeared to provide the dominant discriminatory signal once modeled jointly.

To further characterize the biological and clinical relevance of the five selected protein candidate biomarkers (TEX13B, SUN3, KRT79, HSP60, PUS7), we examined relationships related to disease activity and laboratory measurements with the five proteins and age as explanatory variables (Table 5).

Correlations between the proteins and DAS28 were all weak (all below 0.35 in absolute value, *p* > 0.09), and a model trying to separate high from low disease activity (seventeen vs. eight patients using the 3.2 threshold) reached only an AUC of 0.67. Two individual hints stand out but should be treated cautiously: higher SUN3 was correlated with lower ESR, and higher PUS7 was correlated with lower CRP (rank correlation −0.46, *p* = 0.019). Physical function was linked only to age, rising about 0.028 HAQ–DI points per year.

With respect to anti-CCP, a traditional serological marker for RA, we found that only one HC tested positive for anti-CCP, but despite this seropositivity, the protein-based model correctly classified the sample as being non-RA. Conversely, one HC was incorrectly classified as RA by the protein-based model and tested negative for anti-CCP. After analyzing differences in protein abundance in seronegative RA patients, we found that HSP60 expression was significantly elevated (*p* < 0.001) in this subgroup of RA patients.

To verify if the protein panel was heavily modulated by current medical treatment, we compared protein levels across distinct therapeutic regimens. The expression levels of all five candidate biomarkers showed no significant differences when stratification was made using conventional synthetic disease-modifying antirheumatic drugs (DMARDs), such as methotrexate, or biologic TNF inhibitors, such as Adalimumab.

## 4. Discussion

Together, these results suggest that a multiple-protein-expression profile may serve as a candidate biomarker panel for RA, including seronegative patients. The distinct expression patterns, particularly the upregulation of HSP60, PUS7, TEX13B, and KRT79, along with the downregulation of SUN3, after adjusting for confounding variables such as age justify their further exploration and validation in larger cohorts with undifferentiated arthritis.

This is one of the first approaches conducted in peripheral blood to try to identify a distinctive proteomic profile for candidate biomarkers in RA patients analyzed by clinical, serological, and treatment data. Previous studies with a proteomic approach have analyzed the synovial fluid [15] and serum [16], among other tissues, with some discrepancies in identified proteins compared to ours. However, most of the previous studies focus on early RA or erosive arthritis, and only a few include positiveness to anti-CCP antibodies.

With respect to the candidate biomarkers identified in the present study, although not all proteins were present in every case, their combined differential expression may serve as a candidate biomarker profile to distinguish RA from healthy subjects.

Previous attempts have identified the following as candidate biomarkers: complement components C3 and C4A, fibrinogen, apolipoprotein, alpha 1-antytripsin, TTHM-human transthyretin, angiotensinogen, CATR-tumorigenic protein, human serum albumin, and cytochrome C oxidase [17]. Although some of these proteins have a solid biological link to the inflammatory response in RA, we did not find a significant difference in abundances when comparisons were made between RA and HC patients for these proteins.

Our panel of five protein candidate biomarkers achieved 96% accuracy overall, with only one misclassification per group. Importantly, this classification was independent of anti-CCP status and age, potentially offering a reflection of the underlying inflammatory or tissue-remodeling state. Furthermore, the panel showed a moderate negative correlation with inflammatory markers, specifically SUN3, whereas physical function deterioration was linked to increased age but not to the proteins. This suggests that the discriminative ability of these protein panels may be largely independent of the acute inflammatory state and age, potentially acting as intrinsic state markers of the RA phenotype rather than acute-phase reactants; although while promising, this finding requires validation in larger cohorts to assess reproducibility and clinical applicability.

Among the proposed candidate protein biomarkers, HSP60 is the most important biologically plausible candidate, with well-documented links to autoimmune and inflammatory rheumatic diseases (AIRDs) [18]. HSP60 can act as a superantigen and endogenous danger signal, promoting both pro- and anti-inflammatory responses through pathways involving TLRs and regulatory T cells. The presence of anti-HSP60 autoantibodies in RA and other autoimmune diseases further supports its potential role in both disease pathogenesis and progression [19]. Furthermore, in our study, HSP60 was significantly elevated in seronegative RA patients, which might capture a biological variance that is traditionally missed by standard autoantibody testing.

Keratin 79, primarily studied in the context of hair follicle development, belongs to a broad family of proteins related to keratin function and development, and it has been implicated in epithelial integrity and inflammatory skin diseases as well [20], highlighting the interaction of keratinocytes with immune cells and the role of keratins in cytokine production and barrier function; all of which suggest an indirect link to systemic inflammation, although KRT79-specific data in RA are limited [21].

Protein PUS7 is a member of the pseudo uridine synthase family and lacks direct association with autoimmune disease. However, mechanistic evidence from related proteins such as PUS10 supports the hypothesis that RNA modifications can modulate inflammatory signaling, particularly through tRNA fragmentation and innate immune activation [22]. This opens a possible avenue for future studies exploring the regulatory role of RNA-modifying enzymes in chronic inflammation.

TEX13B remains a less characterized protein, with no current evidence of involvement in immune responses or systemic inflammation. Despite its testis-restricted expression, its presence in the serum of RA patients may reflect aberrant protein expression during systemic inflammation or represent a non-specific byproduct of cellular stress.

Interestingly, SUN3 is known to mediate nuclear–cytoskeletal connections and is essential for cell positioning, adhesion, and migration [23]. These functions may be relevant to RA pathogenesis, where cell movement and immune cell trafficking are critical to synovial inflammation and tissue damage, as well as the resolution of inflammation and tissue repair [24]. Furthermore, SUN3 had a moderate positive correlation with BMI, which underscores the necessity of rigorous age and BMI matching in subsequent large-scale validation cohorts to ascertain that the differential expression is driven by RA pathophysiology rather than underlaying demographic imbalances between the study groups.

Some limitations of the current study include the cross-sectional design, which limits the possibility for a prospective analysis on protein expression changes. The small sample size and the use of label-free untargeted proteomics with relative quantification reduce the robustness of the present study’s conclusions and prevent a typical train–test splitting approach; however, this is a report of preliminary findings; therefore, validation in larger cohorts, preferably in prospective studies, could warrant their use as candidate biomarkers, as well as a more in-depth characterization of their association to clinical, therapeutic, and inflammatory parameters, including the production of autoantibodies. The advantage for future studies is that the candidate proteomic profile was successfully reduced from over 735 proteins to only five.

## 5. Conclusions

The study of proteomics in RA is a powerful area that can have advantages since alterations in the protein profile can be evident before the immune response takes over and induces the production of autoantibodies. Moreover, proteomic platforms offer the possibility to perform multiplex approaches that will more likely address the complexity of the development of an autoimmune disease, such as RA.

In summary, this preliminary report shows a clear, consistent protein signature that distinguishes rheumatoid arthritis from healthy subjects, with SUN3 being the single most informative marker and the five-protein combination reaching a cross-validated accuracy around 0.98. Furthermore, age was disregarded as a confounder, and candidate proteins show no dependable link to an active disease. Future studies should validate these findings in larger cohort studies, preferably in the context of undifferentiated arthritis, before confirming this model for diagnosis.

## Figures and Tables

**Table 1:** Clinical and demographic variables.

Variable	RA (*n* = 25)	HC (*n* = 25)	*p*
Female sex, *n* (%)	23 (92)	22 (88)	0.637
Age, median (IQR)	60 (46–65)	44 (40–48)	0.008
BMI, median (IQR)	26.2 (24.2–30.8)	-	
Disease duration, median (IQR)	7 (4–12)	-	
Functional Class I, *n* (%)	18 (72)	-	
Functional Class II, *n* (%)	5 (20)	-	
Functional Class III, *n* (%)	2 (8)	-	
DAS-28, median (IQR)	3.7 (3.0–4.1)	-	
HAQ–DI, median (IQR)	0.7 (0.1–1.0)	-	
Hemoglobin, g/dL median (IQR)	12.9 (12.0–13.7)	-	
Leucocytes, 103/μL median (IQR)	6.7 (6.2–8.4)	-	
Platelets, 103/μL median (IQR)	299 (244–323)	-	
ESR, mm/h median (IQR)	18.5 (14.5–25.0)	-	
CRP, mg/dL median (IQR)	0.66 (0.3–1.2)	-	
Positive RF, *n* (%)	9 (36)	-	
Positive Anti-CCP, *n* (%)	20 (80)	1 (4)	<0.001
Anti-CCP, IU/mL median (IQR)	23.5 (5.6–106.3)	4.4 (4.4–4.6)	<0.001
Methotrexate, *n* (%)	23 (92)	-	
Corticosteroids, *n* (%)	22 (88)	-	
Sulfasalazine, *n* (%)	12 (48)	-	
Hydroxychloroquine, *n* (%)	9 (36)	-	
Leflunomide, *n* (%)	1 (4)	-	
Infliximab, *n* (%)	2 (8)	-	
Etanercept, *n* (%)	1 (4)	-	
Adalimumab, *n* (%)	1 (4)	-	

RA rheumatoid arthritis, HC healthy controls, BMI, Body mass index; DAS-28, Disease Activity Score-28; HAQ–DI, Health Assessment Questionnaire–Disability Index; ESR, Erythrocyte Sedimentation Rate; CRP, C-Reactive Protein; RF, Rheumatoid Factor; Anti-CCP, Anti-Cyclic Citrullinated Peptides; IQR, Interquartile range.

**Table 2:** Top-ranking discriminative serum proteins in RA vs. HC identified by regularized logistic regression and corresponding univariate differential abundance metrics.

Rank	Protein	Model Coef.	HC (Mean)	RA (Mean)	Raw *p*-Value	FDR (BH)
1	SUN3	−0.0225	750,208	191,694	1.88 × 10^−7^	6.92 × 10^−5^
2	Hsp60	+0.0202	175	140,239	7.59 × 10^−4^	0.0279
3	PUS7	+0.0182	6969	39,495	0.0083	0.1094
4	TEX13B	+0.0181	574,485	1,170,976	6.31 × 10^−4^	0.0258
5	KRT79	+0.0178	275,408	528,489	1.32 × 10^−4^	0.0121
6	ADAMTS13	−0.0160	72,989	5922	0.0060	0.0860
7	B2MG	+0.0159	100,446	213,415	4.38 × 10^−4^	0.0218
8	Loricrin	−0.0158	92,970	3299	0.0091	0.1158
9	PI16	+0.0147	150,571	206,300	0.0026	0.0537
10	SAA2	+0.0146	94,426	1,047,740	0.0010	0.0308

Note: Model coefficients (∣β∣) were obtained from regularized multivariable logistic regression analysis on standardized features. Raw *p*-values were obtained from two-sided Mann–Whitney U tests, and FDR q-values were adjusted across all 735 quantified proteins using the Benjamini–Hochberg procedure (BH). HC healthy controls, RA rheumatoid arthritis; Protein abbreviations: SUN3, SUN domain-containing protein 3; Hsp60, 60 kDa heat shock protein (HSPD1); PUS7, Pseudouridylate synthase 7 homolog; TEX13B, Testis-expressed protein 13B; KRT79, Keratin type II cytoskeletal 79; ADAMTS13, A disintegrin and metalloproteinase with thrombospondin motifs 13; B2MG, Beta-2-microglobulin; PI16, Peptidase inhibitor 16; SAA2, Serum amyloid A-2.

**Table 3:** Candidate protein coefficients obtained from logistic regression analysis for RA as the explanatory variable.

Predictor (Single Variable)	Coefficient (SE)	*p*	OR (95% CI)	AUC
SUN3 (per 1 SD = 369k)	−4.24 (1.39)	0.0023	0.01 (0.00–0.22)	0.930
KRT79 (per 1 SD = 290k)	2.21 (0.73)	0.0024	9.13 (2.19–38.01)	0.816
TEX13B (per 1 SD = 615k)	1.35 (0.44)	0.0022	3.85 (1.63–9.13)	0.782
PUS7 (per 1 SD = 50k)	0.85 (0.42)	0.044	2.33 (1.02–5.33)	0.653
Hsp60 (per 1SD)	77.83 (65.48)	0.23	Very wide	0.731
Age (per 1SD = 11.8 years)	1.41 (0.42)	<0.001	4.10 (1.80–9.35)	0.796
Sex, female	0.45 (0.96)	0.64	1.57 (0.24–10.30)	0.520
Anti-CCP positive	4.56	<0.001	96.0 (10.35–890.6)	0.880

OR odds ratio, CI confidence intervals, AUC area under the curve; Protein abbreviations: SUN3, SUN domain-containing protein 3; Hsp60, 60 kDa heat shock protein; PUS7, Pseudouridylate synthase 7 homolog; TEX13B, Testis-expressed protein 13B; KRT79, Keratin type II cytoskeletal 79; Anti-CCP, anti-cyclic citrullinated peptide.

**Table 4:** Comparison of multiple variable regression model performance.

Model	AUC Apparent	CV 5-Fold	LOOCV	AIC	PseudoR2	Interpretation
A1: Proteins only (five proteins, z-scored)	1.000	0.981	0.978	~12	0.93	Proteins separate RA vs. HC almost perfectly; SUN3 dominates (OR 0.01).
A2: Proteins raw (per 100k/10k)	1.000	0.982	0.982	13.2	0.97	Same as A1 but interpretable units; SUN3 −8.9 per 100k, KRT79 6.4 per 100k.
B1: Proteins + Age + Sex (female)	1.000	0.978	0.978	16.1	0.97	Age-adjusted OR 8.7 per SD but *p* = 0.39 after proteins; SUN3/KRT79 remain strongest; Sex NS.
C1: Proteins + Age + Anti-CCP positive	1.000	1.000	1.000	16.6	0.99	Binary Anti-CCP preserves perfect separation; best CV.

A1 (five proteins, z-scored). Coefficients (regularized L1, MLE diverges due to separation): TEX13B +0.57, SUN3 −15.34, KRT79 +8.15, HSP60 +1.58, PUS7 +1.75. SUN3 and KRT79 drive discrimination. VIF all < 1.24. A2 (raw per 100k/10k): TEX13B +0.09 per 100k (OR 1.09, *p* = 0.81), SUN3 −8.87 per 100k (OR 0.00014, *p* = 0.18), KRT79 +6.42 per 100k (OR 614, *p* = 0.20), HSP60 +1.02 per 100k (OR 2.79), PUS7 +0.37 per 10k (OR 1.45). B1 (Proteins + Age + Sex). An adjusted analysis including age and sex to test if protein signals survive after age/sex adjustment. Age OR was 8.68 per SD (*p* = 0.39, NS after proteins). Sex coefficient adds no incremental value; sex (*p* = 0.65 in univariate). C1: Proteins + Age + Anti-CCP (continuous). Adding serology to the model provides anti-CCP OR ~563 per SD (*p* = 0.20). AUC, Area Under the Curve; CV, Cross-Validation; LOOCV, Leave Out One Cross-Validation; AIC, Akaike information Criterion; RA, Rheumatoid Arthritis; HC, Healthy Controls; OR, Odds Ratio; Anti-CCP, Anti-Cyclic Citrullinated Peptide; NS, non-significant.

**Table 5:** Disease activity and laboratory measurements in association with candidate biomarkers/or increased age.

Outcome	R^2^	Adjusted R^2^	Overall *p*	Most Associated Variable
DAS-28	0.297	0.062	0.32	None (all *p* > 0.05)
ESR	0.329	0.093	0.27	SUN3 (*p* = 0.046)
CRP	0.341	0.121	0.22	PUS7 (*p* = 0.046)
HAQ–DI	0.282	0.043	0.36	Age (*p* = 0.042)

DAS-28, Disease Activity Score-28; HAQ–DI, Health Assessment Questionnaire–Disability Index; ESR, Erythrocyte Sedimentation Rate; CRP, C-Reactive Protein.

## Data Availability

The data that support the findings of this study are available at Zenodo https://zenodo.org/records/19099809, Published 18 March 2026, v1. Due to the clinical nature of the data, this dataset is under Restricted Access. Researchers interested in accessing the full data for validation, meta-analysis, or non-profit academic research purposes must request access through the Zenodo platform or by contacting Dr. Maria G. Zavala-Cerna (maria.cerna@edu.uag.mx). Please include your institutional affiliation and the specific purpose for using the data.

## Abbreviations

The following abbreviations are used in this manuscript:

Anti-CCP	Anti-Cyclic Citrullinated Peptides
ADAMTS13	Metalloproteinase with thrombospondin motifs
BMI	Body Mass Index
B2MG	B-2 microglobulin
CRP	C-Reactive Protein
DAS-28	Disease Activity Score-28
ESR	Erythrocyte Sedimentation Rate
HAQ–DI	Health Assessment Questionnaire–Disability
Hsp60	60 kDa Heat Shock protein
KRT79	Keratin type II cytoskeletal 79
IQR	Interquartile range.
PI16	Peptidase inhibitor 19
PUS7	Pseudo uridylate synthase 7 homolog
RA	Rheumatoid Arthritis
RF	Rheumatoid Factor
SAA2	Serum amyloid A-2
SUN3	SUN domain-containing protein 3
TEX13B	Testis-expressed protein 13B
